# Supplementary material for: Formation Mechanism of High-Purity Ti2AlN Powders under Microwave Sintering
Source: Materials (Basel). 2020 Nov 26;13(23):5356. doi: 10.3390/ma13235356 (PMC7728301; doi:10.3390/ma13235356)
Supplement: Supplementary file 1 [file materials-13-05356-s001.pdf]

materials

Supplementary Materials

Formation Mechanism of High-Purity Ti_2AlN Powders under Microwave Sintering

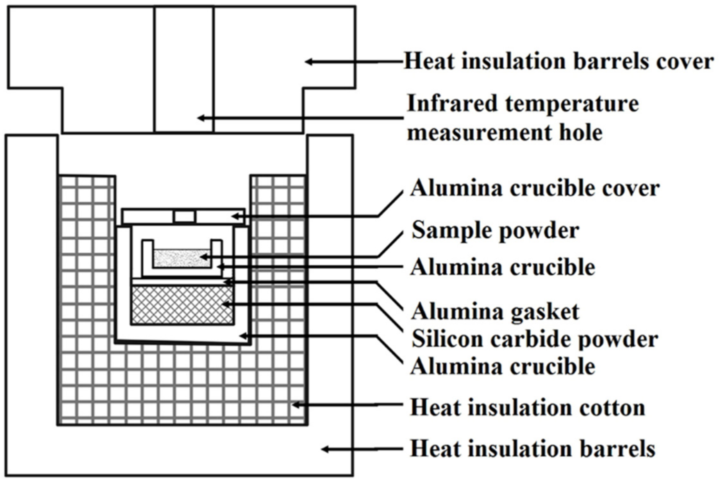

Figure S1. The insulation device of microwave sintering.

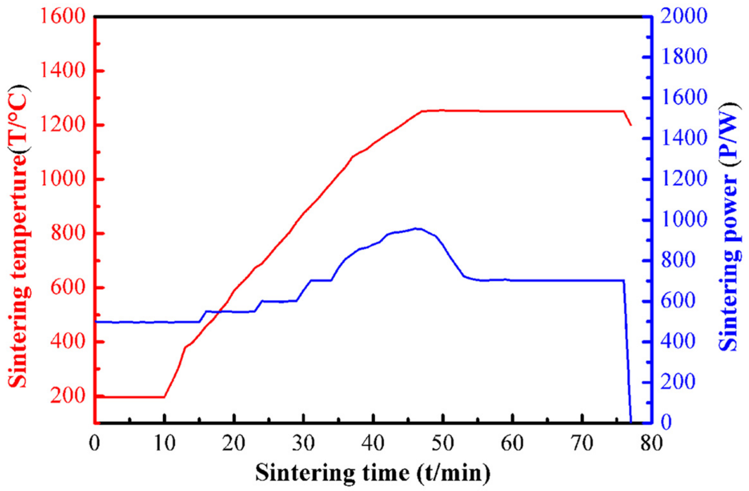

Figure S2. The change curves of temperature and sintering power in microwave sintering.

© 2020 by the authors. Submitted for possible open access publication under the terms and conditions of the Creative Commons Attribution (CC BY) license (http://creativecommons.org/licenses/by/4.0/).
